# United States Veterinary Medical Association

**Published:** 1889-01

**Authors:** 


### UNITED STATES VETERINARY MEDICAL ASSOCIATION.

The semi-annual meeting of this Association will be held in Boston, on Monday, March 18th, 1889. The importance of the association is certainly appreciated theoretically, but practically members are too apt to have their mind on their own "axe," and to forget for the moment the duty they owe to the several score of other members, who have honored them by making them their associates. While the social part of these meetings are very enjoyable and allow one to give vent to acts of self-laudation in professional matters, to compliments justly founded, or of spleen in regard to fellow practitioners, and to reminiscences of amusing days gone-by, yet, do they draw out as they should, the best side, and the greatest scientific ability of the associates. Every member of the Association owes a duty to his profession, to his predecessors who founded his work for him, to other members, most of whom come many miles to meet him, and most of all to himself. His own individual reputation is at stake to be social with those whom he comes to meet, to be honestly accountable for the acts in professional practice which will bring him proper laudation from his colleagues, to be fair in his censure of others and know that charges brought against anyone are based on grounds *pro bono publico* and not for personal ends, to bring up recollections of older members which will stimulate the energies of the newer ones and broaden the field of the veterinarian by the hopes of his own future. Boston has the peculiar reputation of being the "Hub" of learning; let every member of the United States Veterinary Association during the short day of the March meeting acknowledge that important post, to his hosts and colleagues there, and, knowing that he is an important spoke, come prepared to show that radiation is as important to the world as central fires. The Boston meetings have always been good ones, let this one be the best. H.

THE PENNSYLVANIA VETERINARY MEDICAL ASSOCIATION has undertaken the passage of a legislative act in regard to the regulation of the practice of veterinary medicine, which differs essentially from those passed in the last two years in the neighboring States of New York and New Jersey. The new form does not prohibit any one from being employed, or from practicing upon animals in any way, but it does prohibit anyone from calling himself a Veterinarian, or from claiming any analagous title unless he is entitled to it. It affords a much more tangible means of charge for any misdemeanor. A card, a bill head, an announcement of any sort, claiming practice on the part of the delinquent opens him to prosecution with ready proof of his guilt. The moderate limit of five years for non graduates allows any "existing practitioner" ample justice for his *droit d'etre*. It will be to the interest of all agriculturists and stock owners in Pennsylvania to support this bill and work for its passage.

NOTES.—Dr. William Osler, Prof. of clinical medicine in the University of Pennsylvania, has been appointed physician to the Johns Hopkins Hospital, and Prof. of Medicine in the Johns Hopkins University. Dr. Osler took his degree in the McGill

University, Montreal. He subsequently studied in London, Berlin and Vienna, and in 1885 was appointed Gulstonian lecturer in the Royal College of Physicians, London, and in 1886 Cartwright lecturer in the College of Physicians and Surgeons, New York.

Mr. Edward W. Nelson, in his report upon the natural history collections, made in Alaska, speaking of the Eskimo dog, says: "There is a peculiar disease, very much akin to the madness of dogs in lower latitudes, which the Eskimo dog is subject to from Greenland to Behring sea. A dog bitten by another afflicted with this disease becomes afflicted in the same manner in a few days. I made careful inquiry, but could learn of no authentic case where a person had been bitten, although I heard a vague account of a man having died at Pastolik from the effect of such a bite. The natives have the greatest fear of a dog in this condition, yet appear to have a superstitious dread of killing it. Both young and old dogs have the disease, and I have seen a puppy only a few weeks old afflicted with it in the most aggravated form. As a rule the first symptoms appear within four or five days of the time the bite is received. The dog refuses to eat, becomes restless and irritable, his head soon becomes swollen, and his vision is affected. He then has alternating periods of stupid quietness and aimless activity. During the latter he runs blindly about, staggering from side to side, but keeping, with apparent difficulty a nearly straight course until something turns him. When moving about in this way he bites any dog or other living object in his path, and frequently runs blindly into some obstacle from which he starts off in a right angle to his former course. During this time his eyes are fixed and glaring, and his head hangs down as if overweighted, and is slowly swayed from side to side. They are easily avoided, and if kicked out of the way they rarely renew the attack, and never with any spirit. The attack is sometimes preceeded by a hemorrhage from the nose and mouth; in rare cases a dog recovers, but usually they die in one or two days from the time of the first symptoms. During some seasons great numbers of dogs die from this cause."

